# Digital PCR as an Emerging Tool for Monitoring of Microbial Biodegradation

**DOI:** 10.3390/molecules25030706

**Published:** 2020-02-06

**Authors:** Yiqi Cao, Miao Yu, Guihua Dong, Bing Chen, Baiyu Zhang

**Affiliations:** The Northern Region Persistent Organic Pollution (NRPOP) Control Laboratory, Faculty of Engineering and Applied Science, Memorial University of Newfoundland, St. John’s, NL A1B 3X5, Canada; yiqic@mun.ca (Y.C.); my7467@mun.ca (M.Y.); gdong@mun.ca (G.D.)

**Keywords:** biodegradation, digital PCR, monitoring, emerging contaminants

## Abstract

Biodegradation of contaminants is extremely complicated due to unpredictable microbial behaviors. Monitoring of microbial biodegradation drives us to determine (1) the amounts of specific degrading microbes, (2) the abundance, and (3) expression level of relevant functional genes. To this endeavor, the cultivation independent polymerase chain reaction (PCR)-based monitoring technique develops from endpoint PCR, real-time quantitative PCR, and then into novel digital PCR. In this review, we introduce these three categories of PCR techniques and summarize the timely applications of digital PCR and its superiorities than qPCR for biodegradation monitoring. Digital PCR technique, emerging as the most accurately absolute quantification method, can serve as the most promising and robust tool for monitoring of microbial biodegradation.

## 1. Introduction

Our living habitats are detrimentally affected by the accidentally and deliberately released multitudinous contaminants, including both affirmative conventional pollutants and emerging contaminants [1,2,3,4,5]. Microbial-based degradation, occurring with series of chemical and microbial transformations, is the ultimate fate of contaminants with economic and environmental benefits [6,7]. Until now, plenty of contaminants have shown the biodegradability, even the widely used forms of plastics once proven recalcitrant to biodegradation [8,9]. Biodegradation of contaminants is complicated due to the sophisticated interspecies interactions containing cooperation and competition, which will happen in all bioremediation processes like natural attenuation, bio-stimulation, and bioaugmentation [10,11,12,13]. Monitoring biodegradation is then of great importance for understanding the complicated processes and employing appropriate biotechniques for contaminants removal.

Biodegradation initiates when diverse contaminant catalytic enzymes in microbes participate in the redox reactions [14,15]. Parameters including the number of specific degrading microbes (i.e., microbial enumeration), the contaminants genotoxicity (i.e., mutagenicity), and the physiological activity, especially the abundance and expression level of corresponding degrading genes, reflect the biodegradation potential and efficiency [16,17]. For microbial enumeration, the number of survived cultivated bacteria could be calculated based on plate count method [18]. However, this method is with several drawbacks like long time for consuming, high variability, and inability for discerning between strains [19]. Most severely, majority of microbes (more than 99%) could hardly be cultivated in the environment [20], causing results generated from plate count method to be somehow unfaithful. Moreover, the plate-based methods are also widely applied for mutagenicity tests with limitations [21]. For physiological activity analysis, the abundance and expression level of corresponding genes determine the catalytic enzymatic activity [22], which was previously measured through diverse catalytic reactions in vitro [17,23], commanding specific reaction substrates and conditions [24]. Moreover, enzyme assays that can hardly reflect the real circumstance of environmental specimens as ecologically important microbial activities in situ may be low in magnitude, resulting in the boundedness of this method. The flow cytometer method was developed to directly differentiate and determine single cells with regards to size, shape, fluorescence, enzyme activity, etc. [25]. However, flow cytometer requires cells in heterogeneous suspension, making information of aggregated cells unavailable [26].

To resolve these challenges, polymerase chain reaction (PCR)-based techniques work by determining amplified target gene fragments and do not require microbial cultivation, thus emerging for monitoring of biodegradation [27,28]. Behaviors of microbes can be monitored by detecting the occurrence and abundance of specific gene markers. To realize quantification, the PCR-based techniques have been developing from conventional endpoint PCR to real-time quantitative PCR (qPCR) [29]. Recently, the novel digital PCR (dPCR) was reported to be more accurate for absolute quantification of target molecules [30].

The qPCR technique has been widely used for gene quantification. However, limitations still occur in accuracy, sensitivity, precision, and reproducibility [31]. It is proven that dPCR can overcome these limitations with advantages over qPCR in biochemical applications including microbial enumeration [32], low copy target detection [33], environmental DNA detection [34], rare-allele detection [35], minor mutations [36], and analysis of methylated DNA [37]. For biodegradation monitoring, it is essential to accurately quantify microbial performance along with contaminant degradation. Due to the novelty of dPCR, its applications are rarely reported. Besides, few studies clarify the superiorities of dPCR over endpoint PCR and qPCR based on their mathematical theories and technical applications in monitoring of biodegradation. Therefore, in this review, we introduce the three categories of PCR techniques and illustrate the superiorities of dPCR over qPCR for biodegradation monitoring. The dPCR technique is of promising potential on biodegradation monitoring and is expected to be widely adopted in the future.

## 2. Endpoint and qPCR Techniques with Their Applications in Biodegradation Monitoring 

### 2.1. Endpoint PCR

**Figure 1:**
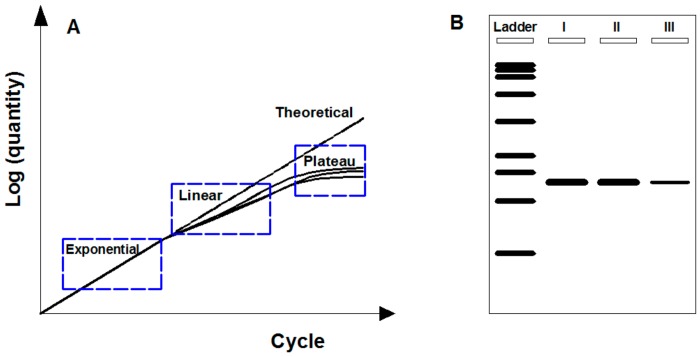
Endpoint PCR-based gene monitoring. (**A**) Description of PCR amplification phases containing both theoretical and practical circumstances. Theoretical amplification: Logarithm of amplified products linear to cycle number. Practical amplification: Consisting of exponential, linear, and plateau phase due to the consumption of reaction reagents. (**B**) Scheme of agarose gel electrophoresis for endpoint amplified products. Target gene can be detected based on size discrimination but not rigorous for quantification.

PCR works by amplifying target fragment of DNA by forward and reverse oligonucleotide primers in multiple cycles of DNA duplexes denaturation, primers hybridization for target sequence, and elongation by DNA polymerase [38]. PCR typically repeats a series of temperature cycles with a doubling of the number of a target fragment generated after each cycle, and theoretically follows exponential amplification (i.e., 2^n^ copies for n cycles). The logarithm of the amplified products is linear to the cycle number (Figure 1A). In practice, due to the consumption of reaction reagents, a basic PCR run mainly consists of three phases (Figure 1A): (1) Exponential phase, specific and precise amplification with 100% reaction efficiency at every cycle; (2) linear phase, slowed amplification with consumed reaction components; and (3) plateau phase, suspended amplification as PCR reagents depleted with no more products generated.

**Figure 2:**
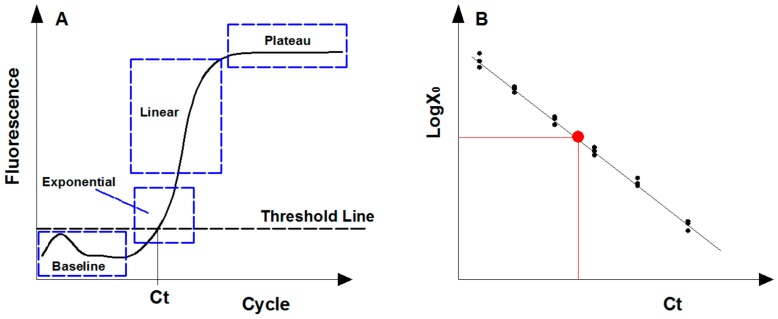
Description of real-time quantitative PCR assay. (**A**) Fluorescence signal levels in four qPCR amplification phases due to the consumption of reaction reagents. (**B**) Standard curve generated by plotting the cycle threshold (Ct) value of diluted standards. The red point represents the target sample that can be calculated following the standard curve.

Endpoint PCR means the amplified products are analyzed at the end of the reaction (i.e., plateau phase) by agarose gel electrophoresis after fluorescence staining. Through this way, comparing with DNA ladder, target genes are detected based on size discrimination. However, endpoint PCR by agarose gel electrophoresis is not rigorous for quantification due to the low gel resolution and variable reaction kinetics [39]. Gel resolution is poor mainly because of the nonquantitative staining dyes (e.g., ethidium bromide), which perform fuzzy identification for low-fold (e.g., less than 10-fold) changes [40]. Variable reaction kinetics occur when the depletion of reaction reagents at different rates after exponential phase for three replicates of a sample, resulting in plateau phase at a different point with different quantities (Figure 1A). In addition, endpoint PCR is semiquantitative by detecting the luminance levels on gel [41]. As shown in Figure 2B, it can roughly detect that the target PCR products of the type III samples were less than those of the types I and II samples but hardly for distinguishing the difference between type I and type II samples.

Endpoint PCR is usually applied for gene detection, molecular cloning, and genotyping through a quick yes or no answer. Besides, endpoint PCR is relatively simple for operation, thus can serve as the pre-step before gene quantification using other accurate techniques by giving the rough results. For biodegradation monitoring, endpoint PCR is applied for verifying the existence of corresponding genes to determine the occurrence of specific microbes’ and contaminants’ biodegradation. For instance, Mauffrey et al. [42] applied endpoint PCR to detect the occurrence of corresponding genes involved in herbicide degradation (i.e., *trz*, *atz*, *phn*, and *puh* genes), suggesting microbial degradation contributing to pesticide dissipation. For anaerobic hydrocarbon degradation, the analogues of genes alkylsuccinate synthases, *assA* [43], and benzylsuccinate synthase, *bssA* [44], were measured by endpoint PCR with degenerate primers, to determine the presence of hydrocarbon degrading bacteria.

Based on endpoint PCR, the denaturing gradient gel electrophoresis (DGGE) and temperature gradient gel electrophoresis (TGGE) techniques were developed to explore the microbial community change [45]. They can separate the PCR amplicons of the same length with differences in GC contents and distributions. For biodegradation monitoring, Luo et al. [46] did the PCR-DGGE for the V3 region of bacterial 16S rRNA to analyze the structure of microbial consortium capable of degrading benzo(a)pyrene. However, because DGGE and TGGE techniques are gel electrophoresis-based, results generated are quantitative and microbes with low abundance can hardly be detected.

### 2.2. Real-Time Quantitative PCR

To satisfy the demands of gene quantification, real-time quantitative PCR (qPCR) was then developed to accurately determine the concentration of target molecular fragments. As its name implies, qPCR means amplification of target DNA fragment is detected in real time during the whole PCR process using fluorescence reporters [47].

For gene quantification, the exponential phase is the optimal point for collecting and analyzing data due to its most efficient amplification, and theoretically the amount of PCR products follows Equation (1) [48]. However, the fluorescence signal of PCR products in early cycles (i.e., baseline phase) is always disordered since the amplification remains at the background level (Figure 2A). To eliminate the background fluorescence signal, the threshold line was applied and set above the background within the exponential phase [49]. The cycle threshold (Ct) is the cycle number at which the amplification plot intersects the threshold line, and the amplified products at Ct cycles follows Equation (2). By logarithmic conservation, the logarithm of initial templates is negatively linear to Ct value, as shown in Equation (3).

The abundance of genes of interest in an environmental specimen are calculated following the calibration curve generated from artificial standards with known quantity (Figure 2B) [50]. Standards used are usually desired nominal gradient diluted genomic DNA (gDNA) with known sequences, plasmids containing target gene constructed commonly through digestion-ligation methods, or artificially synthetic DNA fragments [51]. The 95% confidence intervals (CI) of the target gene concentration by absolute quantification is calculated as Equation (4) [52].

(1) $$X_{n}=X_{0}*{\left(1+E_{n}\right)}^{n}$$

(2) $$X_{\mathrm{Ct}}=X_{0}*{\left(1+E_{n}\right)}^{\mathrm{Ct}}$$

(3) $${\mathrm{LogX}}_{0}={\mathrm{LogX}}_{\mathrm{Ct}}-\mathrm{Ct}*\mathrm{Log}\left(1+E_{n}\right)$$

(4) $$95\%\;\mathrm{CI}=\bar{\mathrm{Ct}}\pm 4.30*\frac{\sigma_{\mathrm{Ct}}}{\sqrt{3-1}}$$

where n is amplification cycle number, $X_{0}$ is initial templates amount, $E_{n}$ is amplification efficiency, $X_{n}$ is amplified products after n cycles, Ct is cycle threshold, $X_{\mathrm{Ct}}$ is amplified products after Ct cycles, $\bar{\mathrm{Ct}}$ is the average of replicated Ct value, and $\sigma_{\mathrm{Ct}}$ is the standard deviation of Ct. Constant 4.30 was calculated from the student’s t distribution when the Ct of replicated qPCR follows normal distribution [53], and constant 3 represents the number of replicate Ct values.

The qPCR technique is widely applied in diverse contaminants’ biodegradation monitoring. For example, Laurie and Lloyd-Jones [54] quantified *phnAc* and *nahAc* genes to monitor the polycyclic aromatic hydrocarbons (PAHs) contaminations in soil. Li et al. [55] found that decreased microcystin degradation correlated to inhibited abundance of *mlrA* gene coding the enzyme responsible for the initial cleavage of cyclic microcystin through qPCR. For the degradation of vinyl chloride, Wilson et al. [56] determined that the abundance of two functional genes (*etnC* and *etnE*) was associated with aerobic degradation in groundwater. However, results generated from qPCR are relative to standard curve and thus could hardly accurately reflect the abundance of target genes. It is promising if we could achieve directly absolute quantification.

## 3. Digital PCR and Its Advantages over Previous PCR Techniques

### 3.1. Digital PCR Systems

The term “digital PCR”, a novel method for the absolute quantification of target nucleic acids without the requirement for standard curves, was first described by Vogelstein and Kinzler [57]. Unlike qPCR, digital PCR hinges on the distribution of template analytes into many replicate microreactors at limiting dilution with most reactions containing one or zero molecules [58]. No reference standards or endogenous controls are needed for dPCR due to its calculation methods, with a sufficiently large number of partitions following Poisson distribution [59].

**Figure 3:**
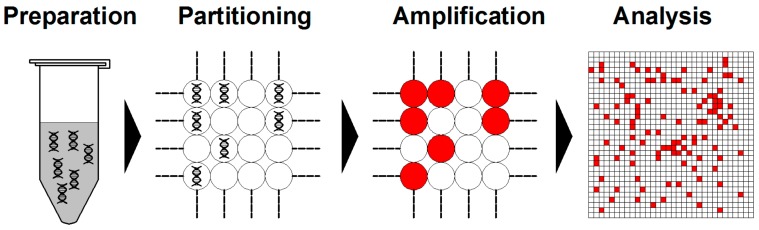
Schemes of typical digital PCR (dPCR) workflow. Generally, dPCR is conducted following the steps of preparation, partitioning, amplification, and analysis.

Briefly, dPCR starts from DNA/RNA extraction in a similar fashion as qPCR. Then, the assembled reaction is partitioned into enormous independent PCR subreactions. PCR amplification is performed to endpoint and the absolute quantification of target molecules is calculated following the Poisson distribution statistical analysis (Figure 3) [60,61].

Poisson distribution enables dPCR for accurate quantification of target molecules [62]. The precision of dPCR increases with an increasing number of partitions. The basic process of dPCR is the random distribution of *m* molecules into *n* partitions. The average number of targets per partition (λ) follows Equation (5), which can be simulated by Poisson distribution as Equation (6). The *m* and C of an unknown sample can be easily calculated from the percentage of empty partitions *P* following Equations (7) and (8). The confidence interval of Poisson distribution is strongly affected by the probability of an empty partition *P* and the average number of targets per partition λ, and the 95% CI for the expected concentration is calculated according to Equation (9) [52,63].

(5) $$\lambda =\frac{m}{n}=C*V$$

(6) λ=−ln P

(7) $$C=\frac{\lambda }{V}=-\frac{\ln P}{V}$$

(8) m=n*λ=−nlnP

(9) $$95\%\;\mathrm{CI}=n*e^{\frac{\ln\left(\lambda \right)\pm 1.96*\sqrt{1-P}}{-\lambda *\sqrt{n*P}}}$$

where λ is average number of targets per partition, m is the number of targets in the sample, n is the number of partitions, C is the sample concentration, V is the partition volume, P is the probability of an empty partition, CI is confidence interval, and 1.96 is a constant for a 95% confidence interval.

**Figure 4:**
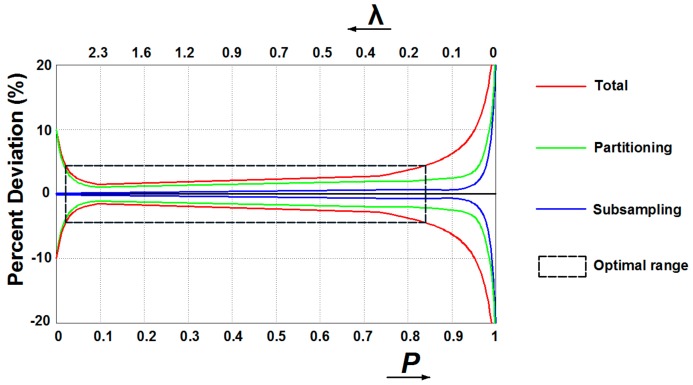
Schemes of assumed total, partitioning, and subsampling errors in digital PCR assays.

In qPCR, some unavoidable inhibitors and amplification competition of templates could seriously affect the detection accuracy since instruments could hardly resolve small differences in emitted fluorescence. Digital assays can overcome these drawbacks as the detection instrument only needs to determine whether amplification occurred in each partition by identifying the individual endpoint fluorescence, making data analysis less dependent on the detector or assay chemistry [59]. However, several sources of variations during dPCR workflow may affect the reliability, among which subsampling and partitioning errors are the most dominant [64]. Subsampling errors arise with the assays when pipetting part volume of the full sample for analysis, resulting in statistical variation between replicates. As modeled by Bizouarn [65], errors dominate when conducting subsampling for the original sample with few targets to detect (i.e., high *P* or low *λ* value) (Figure 4). Partitioning errors occur since the distribution of targets among partitions may differ between each experiment. Dube et al. [66] models the partitioning as a binominal process, with the standard deviation of the probability of an empty partition P propagating to the errors in λ and *P*. Partition errors dominate when very few partitions are empty (i.e., high λ or low P value) and nearly all are empty (i.e., high *P* or low λ value) (Figure 4). Therefore, when there are few targets contained in the original samples (i.e., high *P* or low λ value) subsampling deviations incorporate with partitioning errors, while few partitions are empty (i.e., high λ or low P value) partitioning errors dominate (Figure 4). Considering errors from subsampling and partitioning, preparing samples to the number of copies within the optimal range is the pre-step to improve measurement accuracy.

Two general approaches are commercially adopted for partitioning the initial nucleic acids samples into plenty of individual microreactions, i.e., the chip-based dPCR (cdPCR) and droplet-based dPCR (ddPCR) [67]. For cdPCR, the chip is composed of physically isolated chambers or wells. Applied Biosystems’ QuantStudio^TM^ 3D digital PCR is a commercial representative, with a microchip containing 20,000 microwells and capable of partitioning and allowing separate PCR amplification reactions in these individual microreactors. Detection of the fluorescence of an amplified microchip can thus provide us with the amount of positive and negative reactions for statistical analysis. The second approach, ddPCR, partitions PCR test into sufficiently individual droplets in a water–oil emulsion, with the use of flow cytometry to count positive PCR reactions. Two requirements are needed for the droplet-based reaction. Firstly, the oil should be nonreactive and can form stable microreactors to prevent the diffusion of the reaction reagents. Secondly, appropriate surfactants should be added to stabilize the water–oil interface and prevent oil coalescence. Commercial representative of ddPCR is Bio-Rad’s QX200^TM^ Droplet Digital PCR system.

### 3.2. Fluorescence Reporters in dPCR Systems

Commercially used fluorescence reporters for gene quantification in dPCR system are SYBR Green I and TaqMan assays [68]. SYBR Green I dye is nonspecific fluorescence dye that intercalates with double stranded DNA (dsDNA), while TaqMan-based detection uses a fluorogenic probe specific to target genes.

**Figure 5:**
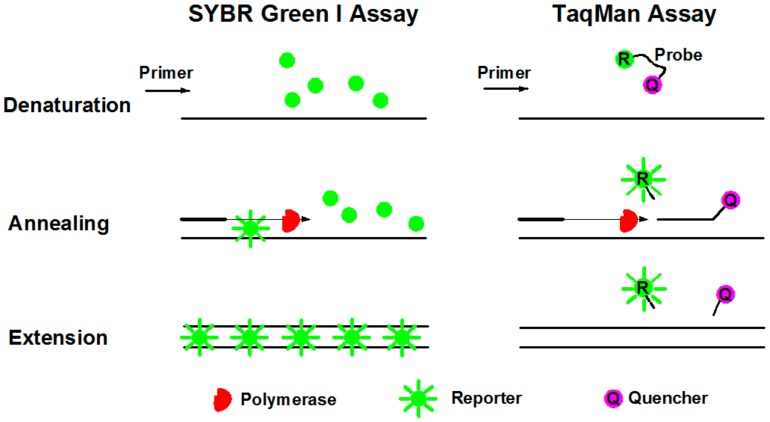
Schematic diagrams of SYBR Green I and TaqMan assays during PCR procedures of denaturation, annealing, and extension.

SYBR Green I dye fluoresces almost 1000-fold greater than its free in solution when it binds to the minor grooves of dsDNA [69]. During denaturation, SYBR Green I dye is released with drastically reduced fluorescence (Figure 5). Annealing and extension generated double stranded PCR products with SYBR Green I binding to it, resulting in a net increase in fluorescence for detection. This method is relatively cost beneficial and easy for operation. The primary disadvantage of SYBR Green I is that it may generate false positive signals since SYBR dye can also bind to nonspecific double stranded DNA sequences. Therefore, it is extremely important to have well-designed primers that do not amplify nontarget sequences, and that melt curve analysis be performed.

TaqMan assay is more specific with an oligonucleotide probe designed for target sequence hybridization [70]. An oligonucleotide probe is constructed of a fluorescent reporter (R) on the 5’ end and a quencher (Q) on the 3’ end (Figure 5). While the probe is intact, the proximity of the quencher dye greatly reduces the fluorescence emitted by the reporter dye by fluorescence resonance energy transfer (FRET). During each annealing and extension, the DNA polymerase cleaves the reporter dye from the probe, leading to the emission of its fluorescence. Due to specific hybridization of the probe, TaqMan assay is more accurate for quantification than SYBR Green I. In addition, probes can be labeled with distinguishable reporter dyes, allowing detection of two distinct sequences in one reaction. The primary disadvantage is the synthesis of specific probes, which require more cost.

## 4. Applications of dPCR for Monitoring of Biodegradation

Biodegradation monitoring via endpoint PCR probably provides us with the occurrence of biodegradation, while qPCR gives us the quantification of these genes but always with inaccurate results. The dPCR can generate accurate results through absolute quantification. In this section, we introduce dPCR application and its advantages over qPCR for monitoring of microbial biodegradation in enumeration of specific degrading microbes, as well as the abundance and expression level regarding functional genes as listed in Table 1.

### 4.1. Microbial Enumeration

Specific degrading microbes directly affect the degradation efficiency. Monitoring the number of these microbes (i.e., microbial enumeration) can then help us understand the biodegradation potential.

Microbial enumeration requires the quantification of specific regions of DNA, with typically used segmental ribosomal markers targeting *16S rRNA* or *23S rRNA* for bacteria, and *18s rRNA* for fungi, internal transcribed spacers (ITS), and other target markers [71,72]. For example, Pornwongthong et al. [73] targeted the *16S rRNA* sequence to quantify the abundance of *Pseudonocardia dioxanivorans* CB1190 during the biodegradation of 1,4-dioxane under the effect of transition metals and organic ligands in the field. Bücker et al. [74] quantified the V4 and V5 regions of *18S rRNA* to determine the abundance of fungi during diesel storage. Besides, during the PAHs biodegradation in the soil, quantification of different ITS regions was used for determining the abundance of each degrading microbe [75]. In addition, Richardson et al. [76] used the B subunit of ribosomal polymerase (*rpoB*) gene as a marker gene to determine the total microbial population in the fuel-contaminated soil as it is present in almost all cells.

To some contents, qPCR cannot meet the demands in microbial enumeration. The first is the detection limit of key microbial indicators during biodegradation. Krolicka et al. [77] quantified *16S rRNA* and the *GyrB* markers to assess temporal microbial variability of oil contaminants in seawater. However, *GryB* gene is a single-copy gene, which is hardly used for quantification by qPCR at low abundance, and they mentioned to employ ddPCR to overcome the challenge. The second limitation is the enumeration calculated using qPCR as calculated from the standard curve, causing the relative but not absolute quantification. Hence, dPCR should be more suitable for microbial enumeration as it can achieve accurate and absolute quantification without calibration.

Recently, some researches have applied dPCR for microbial enumeration during contaminants biodegradation. The dPCR has been successfully used to determine the survival of bioaugmented phthalic acid esters degrading strain *Mycobacterium* sp. YC-RL4 in soil through quantifying the V3-V4 region of *16S rRNA* gene [78]. For dichloromethane dichlorination, Chang et al. [79] used dPCR to quantify the *16S rRNA* gene from 15 key degraders’ genomes to uncover the microbial population dynamics in a given culture. Besides, studies also reported that dPCR was applied in microbial abundance and population dynamics analysis in soil and marine sediment environment [80,81].

Microbial enumeration through dPCR is also suitable to other research, especially for water quality monitoring, i.e., the microbial pathogens detection. United States Environmental Protection Agency (USEPA) recommends using qPCR to quantify enterococci *23S rRNA* as the fecal indicator for water-quality monitoring [82]. However, underestimation always occurs and dPCR could eliminate the weakness caused by qPCR [52]. Besides, dPCR also enables the simultaneous detections of diverse pathogens [83,84] and harmful bloom-forming cyanobacteria [85] through the TaqMan assay.

### 4.2. Functional Gene Abundance Quantification

Microbial enumeration can reflect the abundance of specific degrading microbes. However, it is hard only depending on this to investigate the biodegradation potential since different microbes may harbor different degrading genes and many interactions between species, like horizontal gene transfer always occur during biodegradation. Functional genes are responsible for the synthesis of specific catalytic enzymes involved in biodegradation of contaminants. Therefore, identification and quantification of these functional genes would provide direct information about biodegradation potential in the environment.

Currently, the dPCR is emerging for absolute quantification of these functional genes without standard curve normalization. Kim et al. [86] used 3D chip-based cdPCR technique to evaluate the copy number change of *alkB1* gene responsible for alkanes’ degradation to assess microbial response under seasonal freeze-thaw condition. Dong et al. [87] employed ddPCR to quantify the functional genes involved in the nitrification and denitrification in the natural environment by determining *amoA* gene in ammonia oxidizing bacteria (AOB) and archaea (AOA) and *nirS* and *nosZ* genes in the denitrifiers. In addition, antibiotic-resistant genes (ARGs) are also the emerging contaminants in diverse environmental matrices [88], and researchers applied dPCR for these genes’ quantification in both soil [89] and atmosphere [90] environment to realize accurate measurement more than qPCR.

Horizontal gene transfer always occurs when microbes harboring conjugative or mobilizable plasmids containing genes coding catalytic enzymes are introduced into donor bacteria without contaminants degrading capacity [91]. It was proven that horizontal gene transfer assessment is effective during biostimulation [92] and bioaugmentation [93] monitoring. Through biostimulation, diversity of pollutant-degrading bacteria and the effective transfer of petroleum hydrocarbon degrading genes (i.e., *alkB* and *phnAc*) among resident microorganisms was enhanced for petroleum hydrocarbons degradation [92]. For bioaugmentation, augmented *Rhodococcus* sp. strain p52 harboring catalyzing dioxin degradation genes (i.e., *dbfA* and *dfdA*) located in broad-host conjugative plasmids would transfer the catalytic capacity to other bacteria without degradation ability to enhance biodegradation, while the strain itself would disappear for its unfitness for the environment [93].

However, we have not found any research that utilized the dPCR technique to monitor horizontal gene transfer during contaminants’ degradation. Typically, the ratio of functional gene and target gene (i.e., specific gene sequence for microbial enumeration [93]) abundance is a constant. We usually detect the horizontal gene transfer through measuring the change of this ratio. Traditional qPCR technique measures the two genes separately, resulting in large errors. The accuracy of measurement can improve if we could measure the two genes simultaneously. It is proven that dPCR technique shows the superiority that enables multigene analysis of individual environmental bacteria [94] and many researchers use dPCR for multigene quantifications. For example, Cao, Raith, and Griffith [84] used dPCR for simultaneous quantification of fecal indicators to assess water quality with improved precision and repeatability over qPCR. Moreover, accurate transgene quantification between crop plants was determined by dPCR with high reliability [95]. Therefore, dPCR is more appropriate for horizontal gene transfer monitoring, and applications of dPCR in this area will boost soon.

### 4.3. Gene Expressing Determination

DNA-based quantification always gives a distorted view during biodegradation monitoring, probably overestimating the pollutant degrading ability as it not only presents in active bacterial populations but also in dead microbes. The transcription level of these functional groups of genes coding multicomponent catalytic enzymes would constitute more reliable and accurate biomarkers for the biodegradation monitoring since expression occurs only in metabolically active microbes [96].

Effective biomarkers perform to a relatively high degree of correlation between expression of the functional gene and the rates of contaminants mineralization [97]. Until now, many biomarkers were identified for biodegradation monitoring. For the degradation of 1,2,3- and 1,2,4-trichlorobenzene (TCB), Wagner et al. [98] determined the transcriptions of 32 reductive dehalogenase homologous genes in *Dehalococcoides* stain, suggesting using *cbrA* gene to characterize natural dehalogenation potential. For the degradation of phenoxy acid, the transcripts of *tfdA* gene coding α-ketoglutarate-dependent dioxygenase functions as a molecular marker [97]. The gene *bssA* encoding benzylsuccinate synthase that catalyzes the first step in toluene biodegradation can be employed as a biomarker for biodegradation of toluene [99]. For the emerging contaminant, 1–4 dioxane biodegradation, expression of specific bacterial monooxygenase and dehydrogenase together in *Pseudonocardia dioxanivorans* CB1190 can serve as effective biomarkers to monitoring biodegradation in the environment [96].

Functional gene expression quantification lies in the effectively and specifically messenger RNA (mRNA) detection. Reverse transcription (RT) is the first step that transcribing RNA into complementary DNA (cDNA) for downstream measurement. The conjugated methods, i.e., RT-dPCR or RT-qPCR, are then developed to study gene expression variations. However, RT-qPCR can hardly reflect the actual cDNA amount in the sample and RT operation always introduces the contaminants and inhibitors that impact qPCR analysis. The most acceptable method is employing reference genes, which are assumed constitutively and evenly transcribed across diverse environmental conditions, to normalize and reduce variabilities across samples [100]. Because of the ability of dPCR to absolutely quantify the number of molecules present within a sample without the impact of contaminant inhibition, the use of reference genes and calibration curve seems not obligatory in dPCR. When no effect of measured amount of DNA/RNA is applied to each sample, RT-dPCR can realize directly absolute quantification without normalization [101,102]. For example, during nitrate degradation, Zhang et al. [103] measured the expression level of denitrification-associated genes (i.e., *narG*, *nirK*, and *nirS*) per gram DNA using RT-dPCR and found that reactor with bioaugmented denitrifer strain *Diaphorobacter* could enhance denitrification performance. For nitrogen cycle monitoring, Segawa et al. [104] analyzed the abundance and expression of biomarker genes for nitrogen fixation, nitrification, and denitrification using RT-dPCR, and only gene markers for nitrification and denitrification were highly expressed, indicating this process is the predominant occurrence. In addition, RT-dPCR has lower variability and better reproducibility than RT-qPCR counterpart [105] and the accuracy of RT-dPCR does not rely on amplification efficiency [106]. It thus out-performs RT-qPCR by consistent and precise quantification.

However, in most biodegradation conditions, quality of environmental specimen is highly variable, and all the technical issues associated with the RT step could cause significantly diverse cDNA input for dPCR quantification [107]. The strategy of employing reference genes conducted in RT-qPCR is also beneficial to RT-dPCR application especially in time course experiment [108].

Reference genes applied in RT-dPCR for monitoring of biodegradation is rarely reported. Comparing the superiorities and weaknesses between RT-dPCR and RT-qPCR for diverse applications is the most recent topic, and RT-dPCR is not widely accepted yet. Besides, we found RT-dPCR was applied to monitor lignin degradation by large fungus. Vasina et al. [109] absolutely quantified the expression of corresponding 18 lignin-degrading peroxidases using *tubulin* as reference gene. The expression level of degrading genes was calculated by plotting the absolute concentration of target degrading gene to reference gene. Quantification of biomarkers’ expression relative to references through RT-dPCR will also be adopted for accurate biodegradation monitoring soon.

## 5. Limitations of Existing Applications and Future Perspectives

For biodegradation monitoring, many factors should be taken into account for gene quantification, e.g., the purity and concentration of nucleic acids, the theoretical and practical accuracy, the time and commercial consumption. The dPCR is less affected than qPCR by these factors, with the great potential to be applied in monitoring of microbial biodegradation.

The first factor is the chemical inhibition due to the complexity of environmental samples. Nucleic acids’ extraction is the primary step, which shall unavoidably bring in contaminants to downstream PCR reactions. The qPCR assays are especially vulnerable to contamination since the detection is conducted through the real-time process. It was reported that complex biomolecules, such as humic acid, can significantly inhibit PCR reactions [110]. The dPCR can overcome the shortage due to its endpoint quantification, and the contaminant calcium was reported with less inhabitation for dPCR than qPCR [52]. Besides, dPCR can relieve the effects through increasing number of thermal cycles. Nowadays, widespread emerging contaminants is the global issue. Hence, monitoring the microbial performance for these molecules’ degradation is indispensable. Nevertheless, few researchers discussed whether emerging contaminants may affect the results of gene quantification. From this review, it showed signs that dPCR can be the replaceable toolkit of qPCR for diverse emerging contaminants’ biodegradation monitoring.

The second one is the theoretical and practical accuracy. The qPCR quantification technology highly relies on the reference curve, resulting in relative quantification, while dPCR achieved the absolute quantification through Poisson distribution. For theoretical accuracy, at environmental relevant concentrations, it was proven that dPCR is more precise, with narrow 95% confidence interval, than qPCR quantification [52]. For practical accuracy, results generated from qPCR were relative to calibration curve and were not the actual number of copies in a sample itself. However, different structure types of standard DNA may affect the quantification accuracy. It was proven that qPCR assay is seriously overestimated by using circular plasmid standard as standard when quantifying microalgal *pcna* gene [111]. Besides, the amplification efficiency was instrument dependent among commercially used Eppendorf RealPlex, BioRad CFX96, AB StepOne, AB 7500Fast, Corbett Rotorgene I, and Roche LC480 systems [112], hence qPCR could hardly yield acceptable precision or reproducibility. Errors generated from dPCR were mainly from subsampling and partitioning errors considered into Poisson model, while some other errors, like the partitioning volume [113], should also be taken into the model to improve the accuracy of dPCR application. To standardize experimental protocols and improve the reproducibility of data, researchers should carefully follow the Digital Minimum Information for Publication of Quantitative Digital PCR Experiments (dMIQE) Guidelines [100].

The third one is the low abundant gene quantification. The *16S rRNA* gene is usually used for quantifying the amounts of bacteria. However, multiple copies of this gene are often present in a given bacterium with intragenomic copies differing in sequence, leading to identification of multiple ribosome types [114]. Quantification of single-copy gene (e.g., *rpoB*, *GyrB* markers) is thus promising for microbial enumeration. As a matter of fact, qPCR could hardly quantify low abundant genes, limiting its detection in microbial variability [115,116]. For functional gene monitoring, this circumstance should also be taken in account. The dPCR could absolutely quantify the low-copy number genes, thus the promising future for accurate biodegradation process monitoring.

The fourth one is the commercial cost. Though the qPCR instrument is relatively cheaper than dPCR, it is time consuming and requires standard curve calibration. Standard curve preparation wastes a lot of time and some reagents are extremely expensive. The instrument cost for cdPCR is relative to qPCR instrument (almost $20,000–$40,000), and they are both cheaper than ddPCR (almost $100,000). For cdPCR, the reaction chip can only hold one sample, with the cost of $12 each. If measuring multiple samples, the consumption of chips will raise the monitoring cost. Selecting the appropriate dPCR system strongly depends on the samples numbers. We also believe, with the technology development, the cost will significantly decrease to satisfy the need of the researchers.

With the rising trend of globalized emerging contaminants, microbial biodegradation monitoring technique should be further standardized. The dPCR exhibited the potential for standardizing the data due to its lab independence and absolute quantification. The next generation sequencing (NGS) is the other important technique for microbial community analysis during biodegradation. Results generated from dPCR and NGS can help us better understand the global pollutions and, therewith, the appropriate actions to face these contaminants.

## Figures and Tables

**Table 1:** The dPCR-based monitoring applications and its superiorities over qPCR.

Description	Advantages	Disadvantages	Platform
**Microbial enumeration**			
Quantifying the *16S rRNA* and *GyrB* markers to assess temporal variability of oleophilic bacteria in seawater when facing oil contaminants	Employment of ddPCR to determine single copy number markers like *GryB* gene, which is sometimes undetectable through qPCR		qPCR; ddPCR
Quantifying the V3-V4 region of *16S rRNA* to explore the dynamic change of inoculated *Mycobacterium* sp. YC-RL4 in the soil for phthalic acid esters degradation	Absolute quantification of specific microbes in complex environments with known copy number of *16S rRNA*		ddPCR
Quantifying the *16S rRNA* gene of 15 key degraders to uncover the microbial population dynamics in a given culture for dichloromethane dichlorination	Directly monitoring of single uncultivated bacterial cells and their diversity		cdPCR
Quantifying the *16S rRNA* genes to compare archaea abundance and evaluating the PCR-inhibitory effects of substances from soil and marine subsurface sediments	Accurate and absolute quantification with little inhibitory effects		qPCR; cdPCR
Quantifying the *16S rRNA* using isolated *Cupriavidus* sp. MBT14 and *Sphingopyxis* sp. MD2 as model strains to identify population dynamics in soil	Standard curve unrequired; high sensitivity and efficiency for multi targets measurement; less variability among labs	Time consumption for droplets generation; expensive reaction regents; more steps required than qPCR	qPCR; ddPCR
Quantifying the *23S rRNA* to enumerate Enterococci to assess water quality	Standard curve unrequired; accurate quantification; less affected by inhibitors comparing with qPCR and inhibition could be relieved by dilution		qPCR; cdPCR
Quantifying *rfbE* and* prfA* genes simultaneously to detect pathogenic bacterial contamination (i.e., *E. coli* O157: H7 and *L. monocytogenes*) in water	Simultaneous genes detection via two-color fluorescence probes without cross-assay interference; high accuracy and sensitivity; low detection limit		qPCR; ddPCR
**Functional gene abundance quantification**			
Quantifying the copy number variation of *alkB1* gene to assess the biodegradation potential of nutrient-amended petroleum hydrocarbon-contaminated soil	Absolute quantification without standard curve		cdPCR
Quantifying the copy number variation of *nosZ, nirS* and *amoA* genes in plasmid DNA to assess nitrification and denitrification	Independent of DNA standards	Two measurement bias: (1) plasmid DNA and (2) droplet volume. Linearizing plasmid DNA through restriction and correcting droplet volume could improve reliability and accuracy	ddPCR
Quantifying low copy number variation of antibiotic resistance genes *Sul1* and* qnrB* in soil	High sensitivity; lower detection limit; less affected by environmental DNA templates	Lower range of quantification than qPCR	qPCR; ddPCR
Quantifying 22 antibiotic resistance genes in composting plants’ atmosphere to assess ecological risk of composts	Absolute and accurate quantification without standard curve		ddPCR
Quantifying transgene behavior of *hptII, nptII, bar, ZmUBI1p* genes between crop plants	Accurate and efficient determination of transgene copy number; high reliability		ddPCR
**Gene expression determination**			
Quantifying expressions of *narG, nirK* and* nirS* genes in biofilm samples to assess nitrate degradation in denitrification bioreactor with bioaugmented *Diaphorobacter*	High precision and tolerance to inhibitors and better for complex environmental samples	No reference genes applied may cause inaccuracy	RT-ddPCR
Quantifying expression of *amoA, narG, nirK* and* nosZ* genes to assess nitrogen cycle in cryoconites	Absolute quantification without standard curve		RT-cdPCR
Quantifying expression of *Lip, mnp, vp* genes refer to *tubulin* gene to assess lignin degradation in soil	Absolute quantification; accurate quantification using reference gene; reliable and reproducible measurements of small changes for low abundant cDNA		RT-ddPCR
